# A Constitutional Translocation t(1;17)(p36.2;q11.2) in a Neuroblastoma Patient Disrupts the Human *NBPF1* and *ACCN1* Genes

**DOI:** 10.1371/journal.pone.0002207

**Published:** 2008-05-21

**Authors:** Karl Vandepoele, Vanessa Andries, Nadine Van Roy, Katrien Staes, Jo Vandesompele, Geneviève Laureys, Els De Smet, Geert Berx, Frank Speleman, Frans van Roy

**Affiliations:** 1 Department for Molecular Biomedical Research, VIB, Ghent, Belgium; 2 Department of Molecular Biology, Ghent University, Ghent, Belgium; 3 Center for Medical Genetics, Ghent University Hospital, Ghent, Belgium; 4 Department of Pediatric Hematology and Oncology, Ghent University Hospital, Ghent, Belgium; University of Minnesota, United States of America

## Abstract

The human 1p36 region is deleted in many different types of tumors, and so it probably harbors one or more tumor suppressor genes. In a Belgian neuroblastoma patient, a constitutional balanced translocation t(1;17)(p36.2;q11.2) may have led to the development of the tumor by disrupting or activating a gene. Here, we report the cloning of both translocation breakpoints and the identification of a novel gene that is disrupted by this translocation. This gene, named *NBPF1* for Neuroblastoma BreakPoint Family member 1, belongs to a recently described gene family encoding highly similar proteins, the functions of which are unknown. The translocation truncates *NBPF1* and gives rise to two chimeric transcripts of *NBPF1* sequences fused to sequences derived from chromosome 17. On chromosome 17, the translocation disrupts one of the isoforms of *ACCN1*, a potential glioma tumor suppressor gene. Expression of the *NBPF* family in neuroblastoma cell lines is highly variable, but it is decreased in cell lines that have a deletion of chromosome 1p. More importantly, expression profiling of the *NBPF1* gene showed that its expression is significantly lower in cell lines with heterozygous *NBPF1* loss than in cell lines with a normal 1p chromosome. Meta-analysis of the expression of *NBPF* and *ACCN1* in neuroblastoma tumors indicates a role for the *NBPF* genes and for *ACCN1* in tumor aggressiveness. Additionally, DLD1 cells with inducible NBPF1 expression showed a marked decrease of clonal growth in a soft agar assay. The disruption of both *NBPF1* and *ACCN1* genes in this neuroblastoma patient indicates that these genes might suppress development of neuroblastoma and possibly other tumor types.

## Introduction

Neuroblastoma, a tumor derived from pluripotent neuroblasts, is the most frequent extracranial solid malignancy of childhood. It is remarkably heterogeneous both clinically and biologically, as there is a large variation in tumor progression depending on the age of the patient at the time of diagnosis and the tumor stage [1].

Different genetic abnormalities are frequently observed in primary neuroblastoma tumors and their derivative cell lines. The most frequent aberration is the unbalanced gain of 17q, often due to translocation of the 17q segment to other chromosomes, combined with retention of two copies of the normal chromosome 17. One of the most common translocation partners for the 17q fragment is chromosome 1p, indicating that this translocation confers a selective advantage on cells by disrupting or activating certain genes [2], [3]. As the translocation breakpoints are scattered on chromosomes 1 and 17, there is little evidence for a gene-specific inactivation [4].

Amplification of the proto-oncogene *MYCN*, which occurs in about one quarter of all neuroblastomas [1], is correlated with poor prognosis [5]. Several studies have shown a strong correlation between *MYCN* amplification and deletion of the short arm of chromosome 1 [6], another frequent abnormality in neuroblastomas. Other deletions frequently affect chromosomes 3p and 11q [1] and hint at the presence of tumor suppressor genes (TSGs) in these chromosomal regions.

Previously, we described a *de novo*, apparently balanced, constitutional translocation t(1;17)(p36.2;q11.2) in a neuroblastoma patient [7]. According to Knudson's two-hit hypothesis, constitutional deletions and translocations in patients with specific tumors are often the first indicators of the presence of TSGs. Several lines of evidence lead to the hypothesis that the 1p36 locus harbors one or more TSGs for neuroblastoma. Introduction of the short arm of chromosome 1 in the neuroblastoma cell line NGP results in a differentiated phenotype [8], providing experimental evidence for this hypothesis. Furthermore, constitutional deletions or translocations affecting the 1p36 region have been described in other neuroblastoma patients [9], [10]. Several other tumor types, such as breast cancer, malignant melanoma and colorectal cancer, are also characterized by frequent deletions or translocations of 1p36 [11]. In several types of tumors, including neuroblastoma, the shortest regions of overlap for 1p deletions partly coincide, indicating that the same TSGs may be implicated in different types of tumors [12]. A number of strong candidate TSGs were recently reported for 1p, including the *CHD5* gene [13] and the microRNA-34a [14]. However, deletions in chromosome 1p36 are often very large in neuroblastoma [15], and it is likely that defects in more than one gene may contribute to the malignant phenotype. Expression profiling showed that expression levels of some genes located in the 1p35-36 region were decreased in neuroblastoma tumors and cell lines with 1p deletion as compared to 1p-normal samples [16], [17]. Consequently, it was proposed that the underlying reason for the frequent deletion of this region in neuroblastoma is the decreased expression of several TSGs located in the 1p36 region and not the inactivation of one single classical TSG.

## Methods

### Probe generation

We previously described a cosmid contig spanning the chromosome 17 breakpoint [18]. Nine regions lacking repetitive elements were chosen as probes for Southern analysis and amplified by PCR using PAC RPCI-1 880L8 as template. Probe 6, which is localized on the cosmid contig (GenBank Acc N° AF148647) from position 36,914 to 37,391, was amplified with primers 5′-TCCCAAAAGGCCAGTTTCACAC-3′ and 5′-TCTGCAGGCGTCTCATCTCAAC-3′. Probe 9 is located from position 38,018 to 38,584, and was amplified with primers 5′-GTTGTACCCCCTTGACTTCA-3′ and 5′-GTGCCCAGCAGGAGATTCAAT-3′.

### Southern blotting

Genomic DNA from normal human placenta, the Chinese hamster ovary cell line A/3, and somatic cell hybrids 32-7A and 32-2F53VIII [7] was extracted using standard procedures. DNA was digested with *Bgl*II, *Hin*dIII, *Sac*I, *Xho*I, *Xba*I, *Mco*I, *Pvu*II, *Kpn*I or *Hin*cII. Blotting and hybridization were done according to standard procedures.

### GenomeWalker Libraries and PCR amplification

GenomeWalker libraries were generated according to the manufacturer's instructions (BD Biosciences, Palo Alto, CA, USA). For the 32-2F53VIII cell line, 2.5 μg of genomic DNA was digested with *Pvu*II, *Dra*I, *Stu*I or *Eco*RV. For the 32-7A cell line, 2 μg of genomic DNA was digested with *Dra*I, *Eco*RV, *Pv*uII, *Sca*I or *Ssp*I. Subsequently, the appropriate GenomeWalker adaptors were ligated to the DNA.

To amplify the segments overlapping the breakpoint, long-range PCR was done using Takara LA TAQ mix (Takara, Shiga, Japan) and gene-specific primers GSP1 (5′-CCCCTCAGCTCTGTGCATTTTGTCTA-3′) and GSP2 (5′-CCTCTTGCCCCCACCTAGTGTTTATTT-3′) for the 32-2F53VIII derived libraries, and primers GSP3 (5′-CATAGTGGGGGACATCATGACAGTCAC-3′) and GSP4 (5′-ACACCACCAGCCTCCCTCCATTTCTGA-3′) for the 32-7A libraries.

### RACE analysis

3′RACE was performed with a commercial kit according to the manufacturer's instructions (Invitrogen, Merelbeke, Belgium). Briefly, 2 μg of total RNA isolated from the 32-7A cell line was reverse transcribed using the Adaptor Primer (AP). Two PCRs (PCR-A and PCR-B) were set up using the Unabridged Anchor Primer (UAP) and either GSP1 (5′-GCCCTTATGACTCCAACCAG-3′) or GSP2 (5′-ATTGGCTCATCCTCTCATGTT-3′). Nested PCRs were done using the Abridged Universal Anchor Primer (AUAP) and GSP2 (for nested PCR on PCR-A), or the AUAP and GSP3 (5′-TCCCAGAAAATGAAAGTGATG-3′) primer pair (nested PCR on PCR-B).

To search for chimeric *ACCN1* transcripts, we performed 5′ and 3′RACE analysis on cDNA from the 32-2F53VIII and 32-7A cell lines, respectively, using the Generacer kit according to the manufacturer's instructions (Invitrogen). Briefly, 2 μg of total RNA was reverse transcribed using the supplied oligo-dT primer. In the 3′RACE experiment, we performed a PCR with a GSP (5′-AGCACTACAAACCCAAGCAGTTC-3′) in combination with the supplied 3′ adaptor primer. Nested PCR was done using a GSP (5′-GTGGGCCATGACCTGAAGGATAT-3′) in combination with the supplied 3′ nested primer. In the 5′RACE experiment, we used a GSP (5′-CTTGGATGAAAGGTGGCTCAGACT-3′) together with the supplied 5′ adaptor primer. Nested PCR was performed using a GSP (5′-TGATCTCCAGCCCGTTGCCTGTC-3′) and the supplied 5′ nested primer. Products were cloned in pGEM-T Easy (Promega, Madison, WI, USA).

### mRNA expression profiling by real-time quantitative PCR

For the analysis of the global *NBPF* expression pattern, we determined relative gene expression levels using an optimized two-step SYBR Green I RT-PCR assay employing several reference genes [19]. After establishment of amplification efficiencies of >95% for all primer pairs, the delta-Ct method was used for quantification. SYBR Green I core PCR reagents were obtained from Eurogentec (Seraing, Belgium) and used according to the manufacturer's instructions. Reactions were run on an iCycler (Bio-Rad, California, USA). Gene expression levels were normalized using the geometric mean of four reference genes [19]. Relative expression levels for amplicons in the *NBPF* transcripts were measured in a panel of 31 neuroblastoma cell lines.

For analysis of the *NBPF1* expression pattern we determined the relative gene expression levels by an optimized two-step SYBR Green I RT-PCR assay using the two most stable housekeeping genes in neuroblastoma cell lines [19]. One microgram of total RNA was treated with the RQ1 RNase-free DNase according to the manufacturer's instructions (Promega). Before synthesizing cDNA, treated RNA samples were desalted on Microcon-100 spin columns (Milipore, Bedford, USA). cDNA was synthesized at 54°C using gene-specific primers (*NBPF1*: 5′-CTGGGTGGACTTCGCGTAACTT-3′, *GAPD*: 5′-GGCATGGACTGTGGTCATGAG-3′ and *HPRT1*: 5′-GGTCCTTTTCACCAGCAAGCT-3′) and Superscript III reverse transcriptase according to the manufacturer's instructions (Invitrogen). RT-PCR mixes contained 20 ng template cDNA, LightCycler 480 SYBR Green I Mastermix (Roche Diagnostics GmbH, Mannheim, Germany) and 900 nM forward and reverse primers. Reactions were performed on a LightCycler 480 (Roche Diagnostics) using the following protocol: incubation at 95°C for 5 min, then 50 cycles at 95°C for 10 sec, 66°C for 30 sec, and 72°C for 1 sec.

Primer sequences for the reference genes are deposited in RTPrimerDB, a public database for real-time PCR primers (http://medgen.ugent.be/rtprimerdb/; [20]): *UBC*, *SDHA*, *HPRT1*, and *GAPD*. The primer sequences for the *NBPF* amplicons were as follows: amplicon A: 5′-GATGAATGTCAGGATGCTCTAAACAT-3′ and 5′-CCACCATGCTGACGTTTGTG-3′; amplicon B: 5′-CAGCAGCACATTTCACTCATTAGAG-3′ and 5′-TCACTTGATCCCACCGATGTC-3′; amplicon C: 5′-GAGCTGCTGGAGGCAGTAGAG-3′ and 5′-GGAGTCAGGCTGTTCAAGACAA-3′; amplicon D: 5′-TGGCAACCTGTGCTCAGTCT-3′ and 5′-GGCATGTGCTGCACAGTTATG-3′. The primer sequences for the *NBPF1* amplicon were 5′-GCGAGGCTGCCCGAGCTTCT-3′ and 5′-GACTTCGCGTAACTTCCCATTCA-3′.

All measurements were performed in triplicates and the mean C_p_-value was used in the calculations. Statistical analysis of the groups with *NBPF1* loss and those without loss was performed with the Wilcoxon rank sum test.

### Cell culture and transfection

The colon cancer cell line DLD1Tr21 Tet-On was obtained from Van de Wetering *et al*. [21]. This Tet-On system activates transcription of the gene of interest in the presence of doxycycline (Dox). The cells were cultured in RPMI with 10% fetal calf serum, 100 U/ml penicillin and 100 μg/ml streptomycin. The cDNA for NBPF1-IRES-GFP, fused to an amino-terminal flag tag, was cloned in the pcDNA4/TO vector (Invitrogen). This construct was stably transfected in DLD1Tr21 cells using Lipofectamine reagent (Invitrogen) according to the manufacturer's instructions. Fluorescence activated cell sorting allowed rapid isolation of NBPF1-positive cells, and further subcloning of this population yielded a clone that was more than 90% positive for NBPF1 expression upon induction with Dox (2 μg/ml, Sigma, Steinheim, Germany) for 48 h. As the transfected vector encodes a flag-tagged NBPF1 fusion protein, single colonies were tested for induction of NBPF1 expression by immunofluorescence staining with the anti-flag antibody (Sigma).

### Colony formation in soft agar

Soft agar assays were performed with 6,500 DLDTr21 cells, either stably transfected with a GFP-expressing vector or with the NBPF1-IRES-GFP-expressing vector, both in the presence and absence of Dox. The cells were cultured in six-well dishes in a two-layer soft agar system, with 0.7% (w/v) Bacto agar (Difco, Detroit, MI, USA) on the bottom and 0.35% (w/v) Bacto agar on the top. Liquid medium was placed on top of the agar. During incubation, this medium was refreshed every 2 days to provide a continuous source of Dox. An additional top-agar overlay was given after one week. Experiments were performed in duplicates and the mean value was used for further analysis. Plates were incubated at 37°C with a humidified atmosphere of 5% CO_2_ for 2 weeks and colonies were counted using an inverted microscope. Pictures are taken with a Leica microscope (0.8×magnification) or with an Olympus microscope (4×magnification).

### Ethics

The reported procedures using human samples were fully approved by the Ethics Committee at Ghent University and written informed consent was obtained from each patient since 1983. For patients whose DNA was obtained before 1983, only verbal consent was obtained. Indeed, the Ethics Committee advised that oral consent was sufficient for those patients, and that written consent should not be sought because most of these patients had died, and it was considered more ethical not to raise the matter with the family members again.

## Results

### Identification of rearranged genomic fragments encompassing the constitutional t(1;17) translocation breakpoint

Karyotypic analysis of the lymphocytes of a neuroblastoma patient revealed a *de novo*, constitutional, apparently balanced translocation between chromosomes 1p36.2 and 17q11.2 [7]. Karyotyping of the primary tumor was unsuccessful, mostly due to necrosis. Due to the highly repetitive structure of the chromosome 1p36 region, breakpoint cloning proved to be extremely difficult. For this reason, we constructed a PAC/BAC contig of 1.5 Mbp covering the 17q breakpoint, and by screening a chromosome-17 specific cosmid library we identified three cosmids overlapping the breakpoint [18].

**Figure 1:**
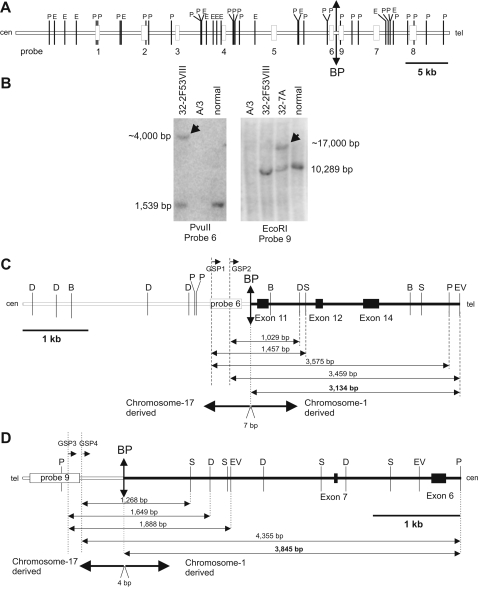
Cloning of the translocation breakpoints of the constitutional t(1;17) translocation. (A) Schematic overview of the sequence of a cosmid contig spanning the breakpoint [18]. Nine repeat-free probes (white boxes) were used in Southern hybridizations. Recognition sites for *Pvu*II (P) and *Eco*RI (E) are shown. (B) Southern blot analysis of genomic DNA of human-hamster cell hybrids containing either the der(1) (hybrid 32-7A) or the der(17) chromosome (hybrid 32-2F53VIII). Left panel: hybridization of probe 6 to *Pvu*II-digested genomic DNA yielded a band of ∼4 kbp in the 32-2F53VIII cell line (arrow) but not in the control samples. Right panel: hybridization of probe 9 to *Eco*RI-digested genomic DNA showed an additional band of ∼17 kbp in the 32-7A cell line (arrow) but not in the control sample. A/3: parental hamster cell line; normal: normal human genomic placenta DNA. (C) Schematic overview of a fragment of derivative chromosome 17. Two primers located in probe 6 (GSP1 and GSP2) were used in combination with adaptor primers to amplify breakpoint-spanning fragments from genomic DNA digested with *Bam*HI (B), *Dra*I (D), *Eco*RV (EV), *Pvu*II (P) or *Stu*I (S). All the resulting fragments contained sequences derived from chromosome 1, with an overlap of 3,134 bp. Seven nucleotides of unknown origin are inserted at the breakpoint site. The location of the t(1;17) translocation breakpoint is indicated by a vertical double-pointed arrow. White boxes represent chromosome 17 sequences, and black boxes represent chromosome 1 sequences. (D) Schematic overview of a fragment of derivative chromosome 1. Two primers located in or nearby probe 9 (GSP3 and GSP4, respectively) were used in combination with adaptor primers to amplify breakpoint-spanning fragments from genomic DNA digested with *Dra*I (D), *Eco*RV (EV), *Pvu*II (P) or *Stu*I (S). All the resultant fragments contained sequences derived from chromosome 1, with an overlap of 3,845 bp. Four nucleotides of unknown origin are inserted at the breakpoint site. The translocation breakpoint and sequences are indicated as in panel (C).

To facilitate the identification and cloning of the t(1;17) translocation breakpoints, genomic DNA from the somatic cell hybrids 32-7A and 32-2F53VIII, the Chinese hamster ovary cell line A/3, and normal human placenta were used for Southern blot analysis. The somatic cell hybrids 32-7A and 32-2F53VIII contain, respectively, the der(1) and the der(17) chromosomes of this neuroblastoma patient [7]. Based on the sequence of the cosmid contig spanning the chromosome 17 translocation breakpoint (GenBank Acc N° AF148647), nine DNA probes free of repetitive sequences were selected (Figure 1A). These probes were used for Southern blot analysis to search for abnormal, patient-specific hybridization bands. Probes 6 and 9 revealed additional bands in the DNA of cell lines 32-2F53VIII and 32-7A, respectively, but not in any of the other DNAs analyzed, indicating that these fragments span the chromosome 17 translocation breakpoint (Figure 1B). Both hybrid cell lines also contain a copy of the normal chromosome 17, resulting in the appearance of a ‘normal’ band in these cell lines.

### Cloning of the constitutional t(1;17) translocation breakpoints in somatic cell hybrids 32-2F53VIII and 32-7A

We used GenomeWalker PCR to clone the breakpoints of the der(17) and der(1) chromosomes, present in the somatic cell hybrids 32-2F53VIII and 32-7A, respectively. For 32-2F53VIII, we used gene-specific primers (GSP1 and GSP2) localized in the probe #6 sequence, together with an adaptor-specific primer (AP1). For 32-7A we used GSP3 and GSP4, localized in or nearby the probe #9 sequence, together with AP1 and AP2, respectively.

Cloning from 32-2F53VIII.

**Figure 2:**
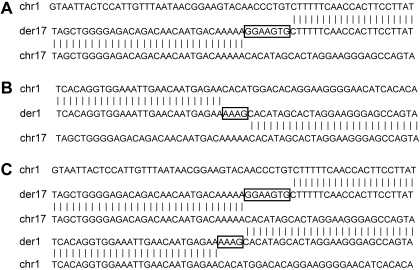
Cloning of the constitutional t(1;17) translocation breakpoints in the cell hybrids 32-2F53VIII and 32-7A. Nucleotide sequences of the der(17) (A) and der(1) (B) regions flanking the translocation breakpoints. (C) Compilation of normal and derivative sequences at the breakpoint sites (NCBI Build 36.1) showing complete preservation of chromosome 17 sequences but not of chromosome 1 sequences.

Primer pair GSP1 and AP1 was used in a first round of amplification. Specific products of 1,457 bp and 3,575 bp were amplified from, respectively, the 32-2F53VIII/*Stu*I and 32-2F53VIII/*Pvu*II GenomeWalker libraries (Figure 1C). With the GSP2 and AP1 primer pair, products of 1,029 bp and 3,459 bp were amplified from, respectively, the 32-2F53VIII/DraI and 32-2F53VIII/EcoRV GenomeWalker libraries. All four 32-2F53VIII-specific products were cloned, and complete sequencing showed overlapping novel sequences derived from chromosome 1 in all clones. In total, we sequenced 3,743 bp flanking the breakpoint region on the der(17) chromosome, of which 3,134 nucleotides were derived from chromosome 1 (GenBank Acc N° AF379607; partly shown in Figure 2A).

Cloning from 32-7A.

Using primer pair GSP3 and AP1, specific products of 1,649 bp and 1,888 bp were amplified from the 32-7A/*Dra*I and 32-7A/*Eco*RV GenomeWalker libraries, respectively (Figure 1D). Primer pair GSP4 and AP2 amplified products of 1,268 bp and 4,355 bp from, respectively, the 32-7A/*Ssp*I and 32-7A/*Pvu*II GenomeWalker libraries. All four 32-7A-specific products were cloned, and complete sequencing showed overlapping novel sequences derived from chromosome 1 in all clones. In total, we sequenced 4,512 nucleotides, of which 3,845 bp were derived from chromosome 1 (GenBank Acc N° AF379606; partly shown in Figure 2B).

### Analysis of sequences flanking the constitutional t(1;17) translocation breakpoints

Previous FISH experiments indicated that the constitutional translocation was balanced [7]. To verify this at the molecular level, we compared the chromosome 17 sequences obtained by GenomeWalker PCR to the sequence of the cosmid contig spanning the der(17) translocation breakpoint. We found that the translocation was indeed balanced on this level (Figure 2C). However, comparison of the chromosome 1 sequences to the human genome reference sequence (NCBI Build 36.1) revealed greater complexity.

**Figure 3:**
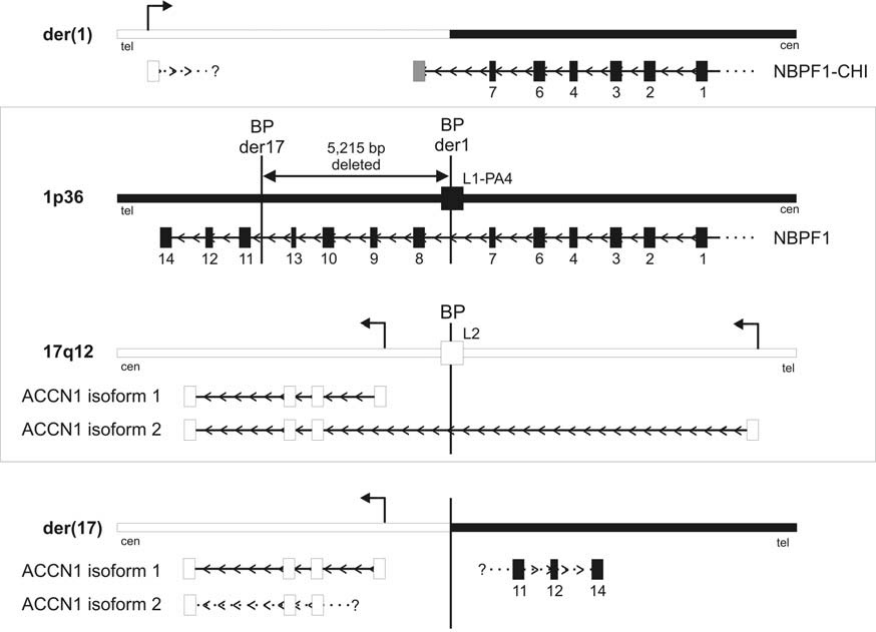
Genomic overview of the translocation breakpoints of the der(1) and der(17) chromosomes. Schematic representation of the normal chromosome 1p36 and 17q12 regions is shown in the central box. On chromosome 1 (black bars) both translocation breakpoints are located in intronic regions of the *NBPF1* gene, which is only partially shown (indicated by the dotted line). The breakpoint of the der(1) chromosome is located in a LINE repeat, shown as a black box labeled L1-PA4 on the genomic region. The black boxes below the genomic sequence represent the exons of the *NBPF1* gene, with the exon type [27] indicated under each box. The translocation resulted in deletion of 5,215 bp. The translocation breakpoint on chromosome 17 (white bars) is located in an *ACCN1* intron and disrupts isoform 2 but leaves isoform 1 intact. Transcription starts are indicated by angled arrows. The breakpoint is located in a LINE repeat, shown as a white box labelled L2 on the genomic region. White boxes below the genomic sequence represent the exons of the *ACCN1* gene. On the der(1) chromosome (top), the *NBPF1* gene is disrupted, giving rise to two chimeric cDNAs (shown as NBPF1-CHI, with the grey box representing the chromosome 17-derived fusion partners). The promoter region and first exon of *ACCN1* isoform 2 are translocated to the der(1) chromosome, but no chimeric transcripts have been isolated yet (shown with the question mark and dotted line). On the der(17) chromosome (bottom), *ACCN1* isoform 1 is unaffected by the translocation, but as the promoter and first exon of isoform 2 are translocated, isoform 2 is probably no longer expressed (shown by the dotted line and question mark). The last three *NBPF1* exons (shown as exons of types 11, 12, and 14) are translocated to the der(17) chromosome. Arrows on the genomic sequence represent promoter sequences, and arrowheads in the transcripts show the orientation of the genes; tel, telomeric end; cen, centromeric end. The figure is not drawn to scale.

In both derivative chromosomes, a few nucleotides of unknown origin had been inserted between the chromosome 1 and chromosome 17 sequences (Figures 1 and 2), a feature frequently observed in reciprocal constitutional translocations [22]. Remarkably, both the chromosome 1 translocation breakpoint cloned from the der(1) chromosome and the chromosome 17 breakpoint were located in LINE repeats (Figure 3). The repeat on chromosome 1 had been classified as type L1-PA4 [23], and the LINE repeat on chromosome 17 as type L2. However, when we compared these two LINE repeats, no significant sequence homology was found. On the other hand, the chromosome 1 breakpoint cloned from the der(17) chromosome was located in a repeat-free region.

We previously reported that *ACCN1* is the closest gene to the 17q breakpoint [18], but after completion of the human genome sequence it became clear that the translocation disrupts one of the isoforms of *ACCN1* (Figure 3). This gene encodes an amiloride-sensitive cation channel [24] with two transmembrane domains [25]. It was shown that the *ACCN1* gene is expressed as two isoforms differing in their 5′UTR and N-terminal domain (Figure 3) [26]. All exons encoding isoform 1 (GenBank Acc N° NM_183377.1) are located proximal to the translocation breakpoint, whereas the first exon of isoform 2 (GenBank Acc N° NM_001094.4) is located distal to the breakpoint. The remaining exons of isoform 2 are shared with isoform 1 and are proximal to the breakpoint. The translocation thus disrupts isoform 2 of the *ACCN1* gene without disturbing isoform 1 (Figure 3). Both isoforms are also found in mouse and rat, strengthening their functional relevance. On the translocated 1p-fragment of the der(17) chromosome, we identified three exons encoding the carboxyterminal domain of the recently described NBPF1 protein. These are shown as exons of types 11, 12 and 14 in figures 1C and 3 (see [27] for numbering of *NBPF1* exon types). *NBPF1* is a member of a novel gene family with an intricate genomic structure [27]–[29]. Analysis of the der(1) sequences proximal to the breakpoint revealed the presence of two exons (shown as exons of types 6 and 7 in Figure 1D and Figure 3) that encode an internal domain of the NBPF1 protein. From this we conclude that the two breakpoints are located within distinct introns of the *NBPF1* gene.

Although the translocation appeared cytogenetically balanced, molecular cloning of the breakpoint sequences revealed a deletion of part of the *NBPF1* gene. Recent reports describing structural variation in the human genome have shown recurrent structural abnormalities in the *NBPF1* locus in normal individuals [30], [31]; we also observed these abnormalities in our analysis of *NBPF1* cDNA clones [27]. Due to this diversity, it is not possible to precisely determine the length of the deleted genomic fragment on chromosome 1 in this patient. However, compared to the human genome reference sequence (NCBI Build 36.1), a fragment comprising at least four exons of the *NBPF1* gene is missing (5,215 bp nucleotides: 16,764,933 to 16,770,147 of chromosome 1).

### Chimeric transcripts resulting from the translocation

Chromosomal translocations within a gene can generate one or two chimeric genes with altered functions. The two open reading frames disrupted in the t(1;17) translocation are encoded by complementary DNA strands, ruling out the possibility of chimeric *NBPF1*-*ACCN1* transcripts (Figure 3). To isolate other chimeric cDNAs resulting from the t(1;17) translocation, we performed RACE (Rapid Amplification of cDNA Ends) analyses on the two hybrid cell lines, each of which contained one of the derivative chromosomes.

We amplified two chimeric *NBPF1* transcripts in the 32-7A cell line (Figure 3). In one chimeric transcript, the type-7 exon sequence was spliced to chromosome-17 sequences located 338 bp distal to the breakpoint, yielding a transcript extended by 295 nucleotides derived from chromosome 17, and an open reading frame extended by 34 additional codons (GenBank Acc No. AF420438). In a second chimeric transcript, the type-7 exon sequence was spliced to chromosome-17 sequences located 8,602 bp from the breakpoint, yielding a transcript extended by 325 nucleotides derived from chromosome 17, and an *NBPF1* open reading frame extended by 11 additional codons (GenBank Acc N° AF420439). The sequences derived from chromosome-17 were not found in other sequences known to be expressed, and were therefore classified as non-expressed sequences. An *in silico* analysis of the chimeric proteins encoded by these transcripts did not reveal any other identifiable domains.

For *ACCN1*, we were unable to detect any chimeric cDNAs in the two hybrid cell lines containing the derivative chromosomes.

### Expression profiling in neuroblastoma cell lines and tumors

**Figure 4:**
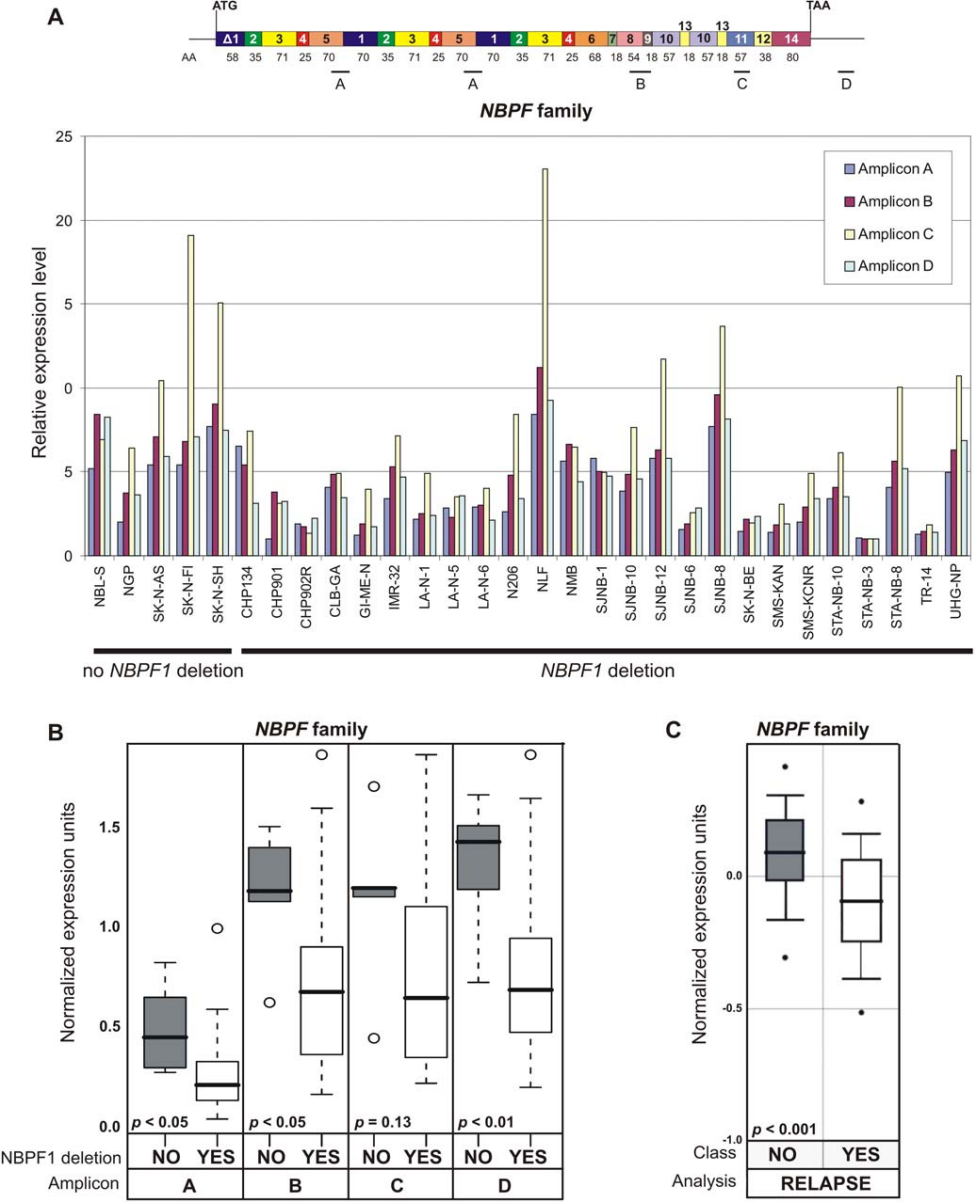
Expression analysis of the *NBPF* gene family in neuroblastoma cell lines and tumors. (A) Real-time quantitative RT-PCR analysis of the *NBPF* gene family in a panel of neuroblastoma cell lines. Four different amplicons on the *NBPF* cDNA were chosen, of which A and C can have several copies in a single *NBPF* transcript [27]. The top panel shows the location of these amplicons in the standard *NBPF1* transcript. Amplicon A is present in two copies in this particular transcript. Expression levels were normalized to four reference genes [19], and the lowest value was set to 1. (B) Neuroblastoma cell lines were grouped according to the *NBPF1* status (heterozygous *NBPF1* deletion or not). For all amplicons, this resulted in a median expression level that was lower in cell lines with *NBPF1* loss, and this difference was significant for amplicons A, B and D (*p*<0.05), but not for amplicon C (*p* = 0.13). (C) Meta-analysis of the *NBPF* expression level in neuroblastoma tumors using the Oncomine database [32]. The reporter used in the array experiment was derived from *NBPF1*, but due to the high sequence identity among the *NBPF* paralogs, this analysis also represents the expression level of all *NBPF* genes. Significantly lower levels of *NBPF* transcripts are observed in tumors that relapse within five years.

A typical feature of a classical TSG is that its function is inactivated in different tumors. This inactivation can be caused by lack of expression due to deletion or promoter silencing. In a first quantitative RT-PCR assay we assessed four amplicons distributed across the *NBPF* transcripts (Figure 4A, top panel). This allowed us to define the expression profile of the complete *NBPF* gene family in neuroblastoma cell lines, as the primers we used bind to regions that show high sequence identity between the different *NBPF* paralogs. Additionally, due to the repetitive nature of the *NBPF* genes [27], some primers can also bind to multiple positions on one transcript. We chose two amplicons that occur only once in a single *NBPF* transcript: on exon types 8-9 (amplicon B) and on the 3′ UTR (amplicon D). In addition, we used two amplicons that can occur in multiple copies in the same transcript: one amplicon on the 5′ repeat exons (amplicon A) and one amplicon on the 3′ repeat exons (amplicon C). In the reference *NBPF1* transcript (GenBank Acc N° NM_017940), amplicon A is present in two copies and amplicon C as one copy (Figure 4A). However, in transcripts from other *NBPF* paralogs, amplicon A can be present as only one copy and amplicon C can be present as multiple copies [27]. These amplicons were analyzed in a panel of 31 neuroblastoma cell lines, some with a heterozygous deletion of *NBPF1* (n = 25) and some with two copies of the *NBPF1* gene (n = 5). As expected, the expression levels of amplicons B and D were comparable (Pearson correlation coefficient 0.95), but in some cases these levels differed considerably from those of amplicons A and C (Pearson correlation coefficient 0.86 for both A–D and C–D). All together, this analysis showed that the expression of all *NBPF* transcripts combined varied widely between different neuroblastoma cell lines: expression of amplicon C in cell lines SK-N-FI, SK-N-SH and NLF was up to fifteen-fold higher than in STA-NB-3. However, the expression level of amplicon B in these three cell lines was only six- to eleven-fold higher than in STA-NB-3. This difference was probably due to increased expression of transcripts containing multiple copies of amplicon C (e.g. *NBPF10*) [27] in the SK-N-FI, SK-N-SH and NLF cell lines as compared to STA-NB-3.

For all amplicons, the median expression level of the *NBPF* family for cell lines with *NBPF1* loss was lower than for cell lines with a normal 1p chromosome (Figure 4B). For amplicons A, B and D, the difference was statistically significant (*p*<0.05) but for amplicon C it was not (*p* = 0.13). Moreover, meta-analysis using the Oncomine database [32] revealed a significantly lower expression level of *NBPF* transcripts in neuroblastomas that relapsed within five years than in those that did not relapse (Figure 4C) [33].

**Figure 5:**
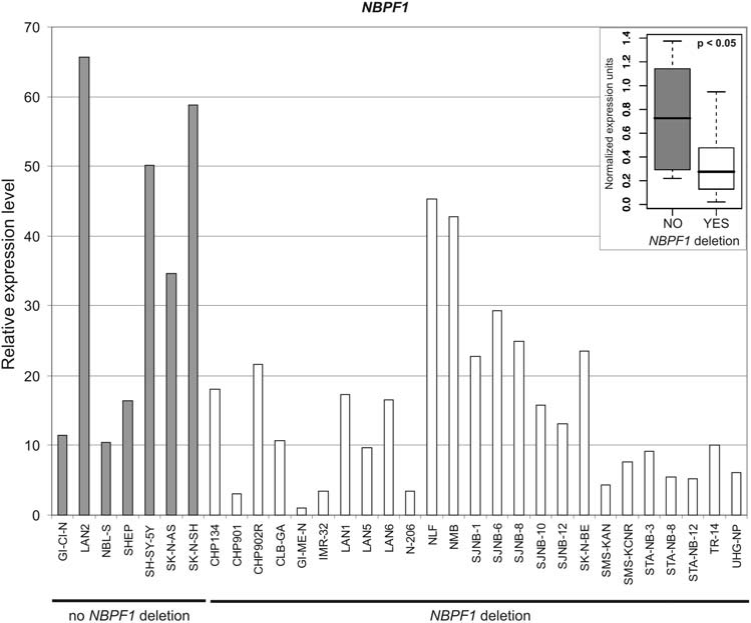
Expression analysis of *NBPF1* in a panel of 32 neuroblastoma cell lines. Expression levels were normalized to two reference genes [19], and the lowest value was set to 1. Inset: relative expression levels of *NBPF1* in neuroblastoma cell lines with normal *NBPF1* status (grey; NO) or with a heterozygous deletion of the *NBPF1* locus (white; YES). Significantly lower *NBPF1* expression is observed in cell lines with *NBPF1* loss (p<0.05).

The abovementioned primers amplified all *NBPF* transcripts, but the constitutional translocation disrupted only the *NBPF1* gene, so we also determined the expression profile of this gene specifically. We designed primers located on the 5′ UTR of the *NBPF1* gene, where it has slightly lower sequence homology with the other *NBPF* paralogs. Specific *NBPF1* amplification was determined by melting curve analysis, which showed a single peak, and by sequence analysis of the amplicon. The expression level of *NBPF1*, determined for a panel of 32 neuroblastoma cell lines, varied widely between the different samples (Figure 5), like it did in the expression analysis of the total *NBPF* family. Interestingly, we did not observe complete loss of *NBPF1* expression in any cell line, as has been described for other genes located in the 1p36 region [16], [17]. Statistical analysis of these data with the Wilcoxon rank sum test revealed a significantly lower expression level of *NBPF1* in cell lines with a heterozygous deletion of the *NBPF1* locus compared to cell lines without *NBPF1* loss (*p*<0.05).

**Figure 6:**
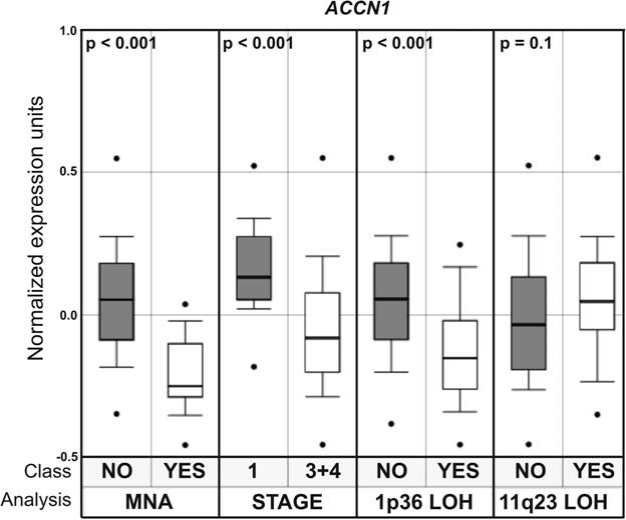
Oncomine expression analysis of *ACCN1* in neuroblastoma tumors. Significantly weaker *ACCN1* expression is observed in tumors with *MYCN* amplification (MNA), in higher stage tumors (stage 3+4), and in tumors with 1p36 loss of heterozygosity (1p36 LOH), but not in tumors with 11q23 LOH.

Our analysis of *ACCN1* (Figure 6) was limited to meta-analysis using the Oncomine database [32]. We observed a significantly weaker expression of *ACCN1* in neuroblastomas with *MYCN* amplification (*p*<0.01) [34] or with 1p36 loss of heterozygosity (*p*<0.01) [34] compared to tumors without these alterations. Additionally, weaker *ACCN1* expression was correlated with higher stages of disease (*p*<0.01) [33]. On the other hand, no difference was observed between neuroblastomas with or without 11q23 loss of heterozygosity (LOH) (*p* = 0.10) [34].

### *NBPF1* expression suppresses anchorage-independent growth

**Figure 7:**
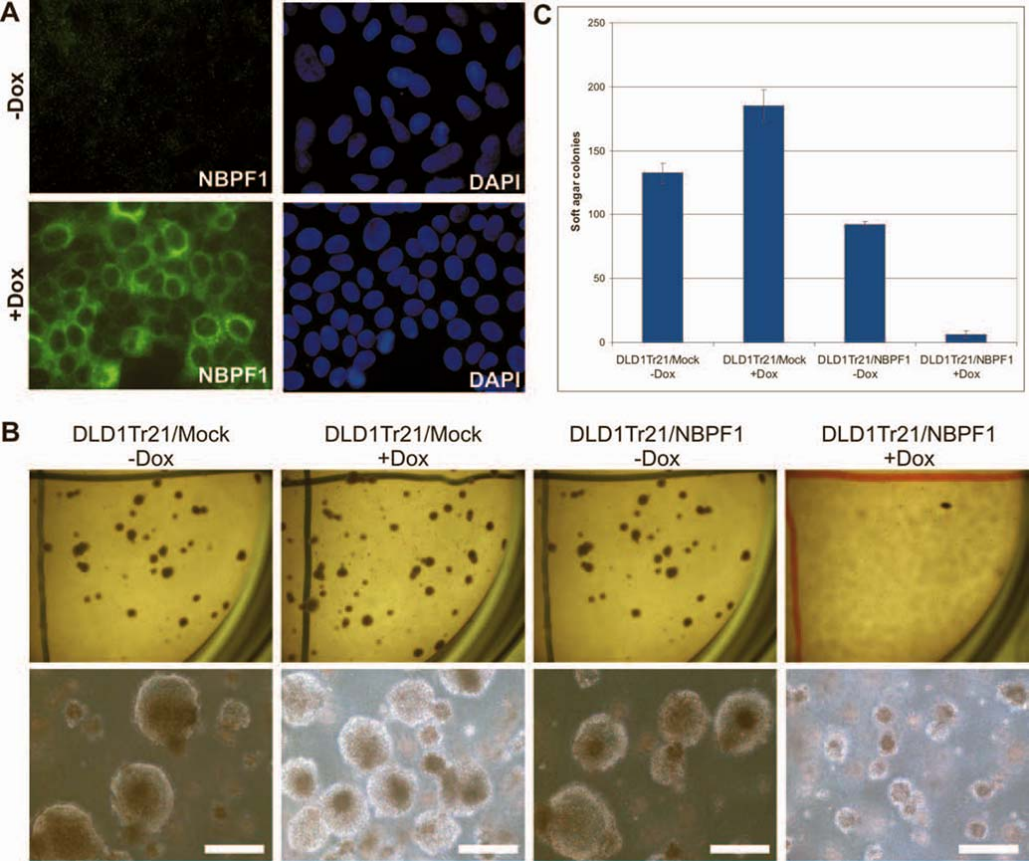
Induced expression of *NBPF1* in human tumor cells impairs colony formation in soft agar. (A) DLD1Tr21/NBPF1 cells express flag-tagged NBPF1 in the cytoplasm, but only when Dox is added to the medium (left panels). DAPI staining was used to visualize nuclei (right panels). (B) DLD1Tr21/NBPF1 and DLD1Tr21/Mock cells were seeded in soft agar in six-well plates in the absence or presence of Dox and then incubated for 2 weeks. NBPF1 induction induces a severe decrease in the ability to form colonies on soft agar. Pictures in the top panel were taken at a 0.8×magnification and those in the bottom panel at a 4×magnification. Scale bars indicate 200 μm. (C) Quantification of the soft agar colonies (mean±standard deviation for two experiments). Only colonies larger than 100 μm were counted. Induction of NBPF1 expression by adding Dox results in a severe decrease in the number of colonies in comparison to the control setting (Mock) or the non-induced (-Dox) cells.

The DLD1Tr21 cell line is a human colon cancer cell line expressing high levels of the tetracycline repressor protein, resulting in a tight control of the expression of the gene of interest under control of a responsive tetracycline operator element [21]. This reliable cell system was stably transfected with an expression vector harboring an inducible flag-tagged full length *NBPF1*. As shown in Figure 7A, the addition of doxycyclin (Dox) to the cell cultures for 48 h resulted in the expression of NBPF1 in the cytoplasm of the cells.

To determine whether overexpression of NBPF1 influences anchorage-independent growth, we compared the ability of DLD1Tr21/mock and DLD1Tr21/NBPF1 cells to form colonies in soft agar in the absence and presence of Dox. NBPF1-expressing cells formed significantly fewer colonies than the control cell lines or the non-induced DLD1Tr21/NBPF1 (figures 7B and 7C). These data indicate that overexpression of NBPF1 strongly inhibits the ability of this human cancer cell line to form colonies in soft agar.

## Discussion

Constitutional chromosomal abnormalities in patients bearing specific tumors have often been used to clone new TSGs. Here, we describe the breakpoint cloning of a constitutional translocation t(1;17)(p36.2;q11.2) present in a neuroblastoma patient. Aberrations in the chromosomal regions involved in this translocation are recurrent in neuroblastoma, and so we hypothesized that this translocation predisposed the patient to the development of neuroblastoma. We cloned both translocation breakpoints and identified on chromosome 1 a novel gene that is interrupted by the translocation. This gene is a member of the recently described *NBPF* gene family, which comprises 22 genes with highly repetitive exon types [27]–[29]. As these genes are located in regions of segmental duplications on chromosome 1, they show high sequence homology in both coding and non-coding regions.

Unbalanced translocations between chromosomes 1 and 17 are common in neuroblastomas, but currently it is unclear whether there is a link between these somatic translocations and the constitutional balanced translocation described here. Only a few of the translocation breakpoints have been cloned [35], [36], and no unifying underlying mechanisms for the translocations have been proposed. Two groups reported increased frequency of chromosome 11q translocations in regions of segmental duplications [37], [38]. In this respect, is striking that the chromosome 1 breakpoint in the t(1;17) translocation described here is also localized in a region of segmental duplication.

*NBPF1* is located on human chromosome 1p36. Though it is located outside of the smallest region of overlap defined by a large-scale analysis of neuroblastoma tumors [15], at least one copy of the region encompassing the *NBPF1* gene is deleted in most neuroblastomas with 1p deletion. Tumor material from the neuroblastoma patient with the constitutional translocation was not available for genetic analysis, so we cannot exclude the possibility that the der(17) was lost in the tumor, leading to loss of the 1p segment distal to the translocation breakpoint. All genes located distal to the *NBPF1* gene would therefore show LOH in the tumor, including a number of recently identified TSGs located in this region [13], [14]. Alternatively, as our *NBPF1* expression profiling revealed a decreased expression in neuroblastoma cell lines with a heterozygous deletion of the *NBPF1* locus, it is also possible that *NBPF1* acts as a haploinsufficient gene. The expression pattern of *NBPF1* is reminiscent of the expression analysis of other genes located in the 1p35-36 region, where 15–20% of the genes showed a lower expression level in neuroblastoma tumors and cell lines with 1p loss of heterozygosity [17], [39]. It has been proposed that the combined diminished expression of these genes, rather than the inactivation of one single classical TSG, could cause the unfavorable outcome associated with 1p deletions in neuroblastoma. In addition to analyzing specifically the *NBPF1* gene, we also analyzed the complete *NBPF* gene family in neuroblastoma cell lines; most amplicons showed a significantly lower expression level in neuroblastoma cell lines with *NBPF1* loss. In these cell lines the deletion was limited to *NBPF1* and *NBPF3*, and the other *NBPF* members, located on chromosome 1q21, apparently cannot fully compensate for the decreased expression of the two deleted *NBPF* genes. Our qRT-PCR analyses were complemented by a meta-analysis using the Oncomine database [32]. These analyses showed decreased *NBPF* levels in neuroblastomas that relapsed within five years. During our previously reported analysis of the *NBPF* family, we detected structural variation in the *NBPF1* gene in different individuals [27]. Similar results were reported in large-scale studies investigating copy-number variation between people [30]. These structural variants of *NBPF1* and its paralogs should be taken into consideration when choosing amplicons and analyzing the expression levels of these genes in different cell lines.

Furthermore, expression of NBPF1 in a human colorectal cell line severely suppressed soft agar colony formation, demonstrating that NBPF1 might act as a TSG, at least in colon cancer. So far, technical difficulties have prevented us from testing this hypothesis in neuroblastoma cell lines. As colorectal cancer is also characterized by frequent deletions or translocations of 1p36 [11], we believe that our present data is a first step in the elucidation of the potential tumor suppressive properties of *NBPF1*. The underlying mechanism for this *NBPF1* activity is currently unknown.

*ACCN1* encodes an amiloride-sensitive cation channel and can be expressed as two different isoforms differing in their aminoterminal domains [26]. The constitutional translocation in the present patient truncates the longer isoform. Interestingly, restoration of surface expression of this isoform in glioma cells reduces cell growth and migration [40], classifying it as a potential tumor suppressor. Meta-analysis of *ACCN1* expression in neuroblastomas showed a correlation between 1p36 LOH and decreased levels of *ACCN1*, but no such correlation was observed between 11q23 LOH and the expression level of *ACCN1* [34]. LOH of 1p36 and of 11q23 are both associated with high-risk disease, but only 1p36 LOH is associated with *MYCN* amplification [6]. Another report showed a decreased expression level of *ACCN1* in neuroblastomas with high telomerase activity [41]. Knockout mice defective in both *ACCN1* isoforms were viable, fertile, and showed no morphological or behavioral abnormalities, but did indicate that *ACCN1* has a role in retinal function [42]. No data have been reported on tumor incidence or tumor aggressiveness in these mice. The most frequent chromosomal abnormality in neuroblastomas is gain of chromosome 17 [43], which appears to conflict with a potential tumor suppressor function for *ACCN1* in neuroblastoma. However, most 17q breakpoints are distal to *ACCN1*, and it has been suggested that the 17q12 region might contain a gene that suppresses neuroblastoma [44]. These data warrant further investigation into the role of *ACCN1* in neuroblastoma. Additionally, it remains possible that the translocation analyzed here affects an unidentified gene in the *ACCN1* intron in which the translocation breakpoint lies. In this region we observed two putative promoter sequences, as predicted by FirstEF [45], and a small number of ESTs not annotated to a known gene and devoid of a meaningful open reading frame (data not shown). It is not clear whether these sequences have a function or are merely the result of random transcriptional activation.

In conclusion, we describe the molecular cloning of both translocation breakpoints in a neuroblastoma patient with a constitutional t(1;17) translocation. Genes overlapping both translocation breakpoints were identified, namely *NBPF1* on chromosome 1 and *ACCN1* on chromosome 17. Further research might reveal the role of the disruption of both of these genes in the development and progression of neuroblastoma.
